# Analyses of Developmental Rate Isomorphy in Ectotherms: Introducing the Dirichlet Regression

**DOI:** 10.1371/journal.pone.0129341

**Published:** 2015-06-26

**Authors:** David S. Boukal, Tomáš Ditrich, Dmitry Kutcherov, Pavel Sroka, Pavla Dudová, Miroslav Papáček

**Affiliations:** 1 Department of Ecosystem Biology, Faculty of Science, University of South Bohemia, České Budějovice, Czech Republic; 2 Department of Ecology and Biosystematics, Institute of Entomology, Biology Centre of the Czech Academy of Sciences, vvi, České Budějovice, Czech Republic; 3 Department of Biology, Faculty of Education, University of South Bohemia, České Budějovice, Czech Republic; 4 Department of Entomology, Faculty of Biology, St. Petersburg State University, St. Petersburg, Russia; 5 Department of Zoology, Faculty of Science, University of South Bohemia, České Budějovice, Czech Republic; University of Connecticut, UNITED STATES

## Abstract

Temperature drives development in insects and other ectotherms because their metabolic rate and growth depends directly on thermal conditions. However, relative durations of successive ontogenetic stages often remain nearly constant across a substantial range of temperatures. This pattern, termed ‘developmental rate isomorphy’ (DRI) in insects, appears to be widespread and reported departures from DRI are generally very small. We show that these conclusions may be due to the caveats hidden in the statistical methods currently used to study DRI. Because the DRI concept is inherently based on proportional data, we propose that Dirichlet regression applied to individual-level data is an appropriate statistical method to critically assess DRI. As a case study we analyze data on five aquatic and four terrestrial insect species. We find that results obtained by Dirichlet regression are consistent with DRI violation in at least eight of the studied species, although standard analysis detects significant departure from DRI in only four of them. Moreover, the departures from DRI detected by Dirichlet regression are consistently much larger than previously reported. The proposed framework can also be used to infer whether observed departures from DRI reflect life history adaptations to size- or stage-dependent effects of varying temperature. Our results indicate that the concept of DRI in insects and other ectotherms should be critically re-evaluated and put in a wider context, including the concept of ‘equiproportional development’ developed for copepods.

## Introduction

**Fig 1:**
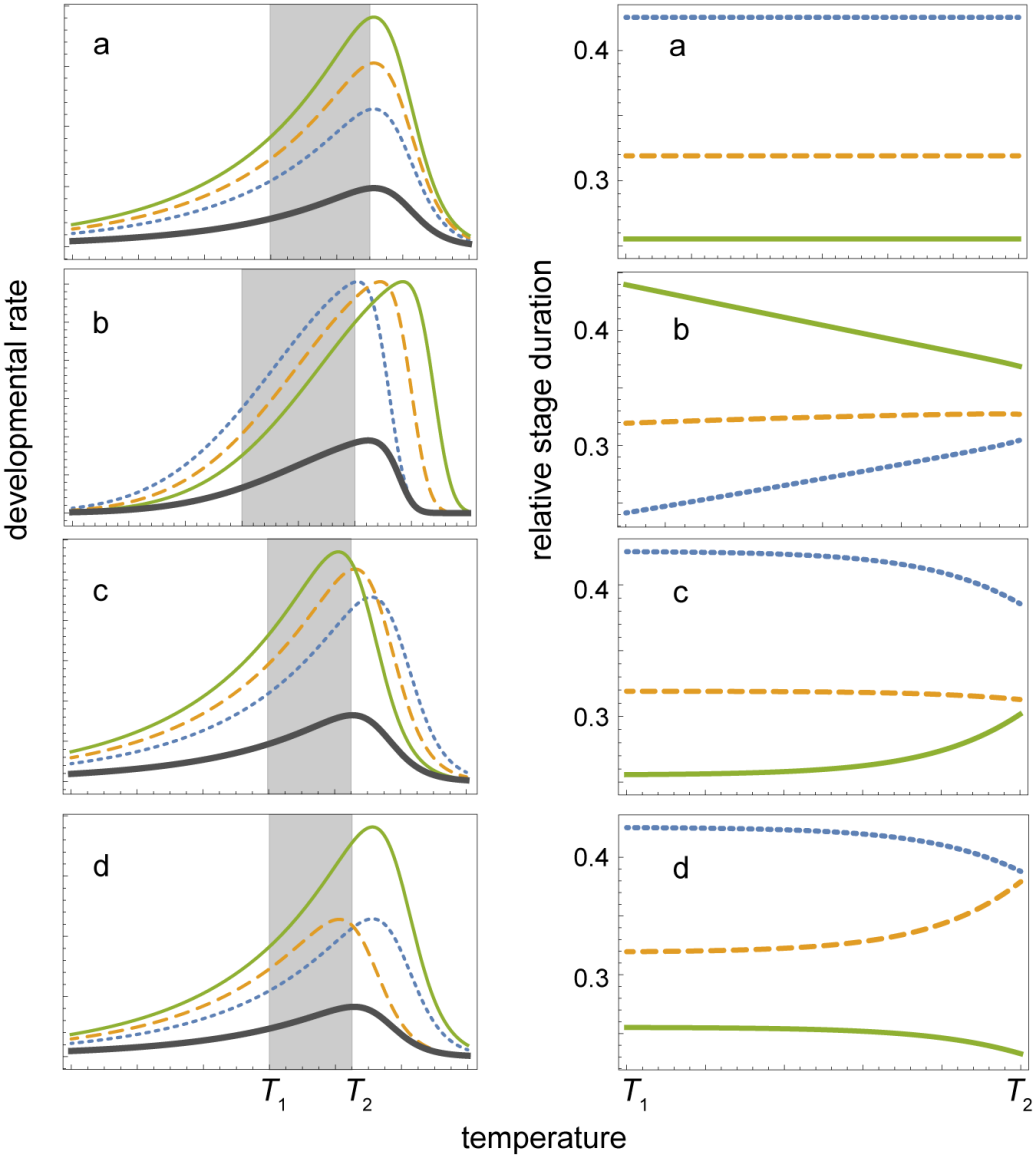
Possible mechanisms leading to DRI violation. Left column shows development rates of three hypothetical life stages, early (blue dotted line), intermediate (orange dashed line) and late (green line), and the overall rate of development (thick grey); grey rectangles highlight the thermal range (*T*_1_, *T*_2_) in which the overall rate of development increases almost linearly with temperature; time units are omitted. Right column shows the corresponding relative duration of each stage in the (*T*_1_, *T*_2_) range. (a) DRI; (b) violation of DRI due to shifted, stage-dependent temperature optima; (c) violation of DRI due to progressively limiting, temperature-dependent metabolic scope for growth in individual stages; (d) violation of DRI due to limiting metabolic scope for growth in the intermediate stage. Even more severe DRI violation outside the (*T*_1_, *T*_2_) range in panels (b)–(d) is omitted for clarity. See text for further details.

Temperature drives development of ectotherms. Within an ecologically relevant thermal range, the entire development from egg to adult is faster at higher temperatures because they enhance the development rate through increased metabolic rate [1]. Moreover, previous studies found that temperature does not affect the proportion of time spent in any given non-diapausing developmental stage, at least within the thermal range for which the rate of development increases linearly with temperature (Fig 1a). This concept is called ‘equiproportional development’ (EPD) in the copepod literature, where it was first described by Corket and McLaren [2] and formalized by Corket [3], and ‘developmental rate isomorphy’ (DRI) in the insect literature, where it was first proposed by van Rijn *et al*. [4] and formalized by Jarošík *et al*. [5]. Although EPD has a longer history, considers a wider thermal range beyond the near linear part of the response in development rate and has been extended to situations when food is limiting [6–8], we primarily use the term DRI as our focus in this paper is on temperature-dependent development in insects under conditions of food satiation.

DRI has important consequences for the effect of temperature on individual development. For example, DRI implies that lower developmental threshold (LDT) temperature, at which the development should be completely arrested, is equal for all stages in a species’ ontogeny [5,9,10]. DRI appears to be common in insects and mites: in the largest study to date, Jarošík *et al*. [5] found DRI in approximately two thirds of more than 420 populations belonging to almost 350 species, and reported that data from most remaining populations violated the DRI concept only to a small extent. Additional studies suggested that relative duration of a particular developmental stage may be a fundamental life history invariant that is independent not only of temperature, but also of host plants, geographical origin of the population, and other factors [5,9], and reported that DRI prevails in other vertebrate and invertebrate ectotherms beyond arthropods [11]. This contrasts with the lack of a clear pattern in temperature dependence of the ratio of total copepodid to total naupliar duration in papers dealing with EPD in copepods [6].

Widespread validity of DRI would have important consequences for pest monitoring and forecasting [11] as well as for the presumed effects of climate change on populations. If all ectotherms more or less complied with DRI, models predicting the impact of climate change on ontogeny and biotic interactions could be simplified because all individuals of a given species would respond similarly to changes in temperature, at least when other factors such as food availability would not constrain their development and growth rates. However, as different species respond markedly differently to temperature [12], it is equally plausible that similar differences may arise during ontogeny and lead to DRI violation.

So how strong is the evidence for the ubiquity of DRI? Analysis of marine pelagic copepod data based on a linear mixed-effect model found that development rates of eggs, nauplii and copepodites scale differently with temperature when all data are pooled and species, study, sex, and stage are treated as random effects [8]. This study has provided a strong indirect support to the idea that DRI may not hold in some groups. All other statistical methods currently used to evaluate DRI are based on analyses of data for individual species. They have their strengths but also potentially serious caveats discussed below.

Most importantly, proportions of time spent in different instars represent compositional data, for which dedicated statistical methods exist [13] but have not been used in DRI or EPD studies. To address this issue, we introduce a new method of DRI analysis that takes into account the inherent proportional structure of the data. The underlying Dirichlet distribution is a continuous multivariate probability distribution generalizing the beta distribution for *n* ≥ 2 variables. The so-called common parameterization of a Dirichlet distribution consists of a vector *α* = (*α*_1_, …, *α*_n_) of positive real numbers, where $\alpha_{0}=\sum_{i=1}^{n}\alpha_{i}$ provides a ‘precision’ parameter (higher *α*_0_ means less variation around the expected value) and *α*_i_/*α*_0_ specifies the mean value of the *i*-th variable, such as relative duration of the *i*-th developmental stage; see [14,15] for details. Dirichlet regression directly evaluates DRI for individual-level data coming from a Dirichlet distribution: it is analogous to linear regression because it estimates the link between any explanatory variables such as temperature and the common parameterization of the distribution.

As a case study, we apply Dirichlet regression to individually resolved data on temperature-dependent development of four terrestrial and five (semi)aquatic insect species. Although a full review of possible causes of DRI violation is outside the scope of our paper, we argue that mechanistic insights [8] can challenge the presumed widespread validity of DRI with alternative testable hypotheses. Here we use the analyses to propose and examine two previously untested mechanisms that could explain the violation of DRI in non-extreme situations, i.e., the thermal range within which the total development rate scales linearly with temperature. This approach is conservative and allows for direct comparison with previous DRI studies, although it could neglect informative DRI violations at suboptimal thermal conditions.

The first such mechanism derives from classical life history theory [16–18]: in temperate regions, different instars of univoltine species may encounter predictably seasonal environment with different temperatures favouring life histories with non-isomorphic rates of development. More precisely, relatively slower development of late developmental stages at lower temperatures could be adaptive if thermal preference curves of individual instars could evolve to track the seasonal trends in temperature by shifting their maxima (Fig 1b). The second mechanism follows recent advances in metabolic ecology: with increasing temperatures, larger individuals can have a progressively narrowing scope for growth due to different allometric scaling of energy intake and expenditure [19,20]. Consequently, larger individuals would develop relatively slowly at higher temperatures. This could be either the latest developmental stage if all stages are actively feeding (Fig 1c), or the intermediate stage if the data are resolved into eggs, larvae and pupae (Fig 1d), because the pupal stage does not feed and draws energy from the reserves accumulated by the larva. Last but not least, the amount of dissolved oxygen declines strongly with water temperature. Stage-specific development rates of aquatic and terrestrial ectotherms may thus respond differently to temperature, similar to the differences in intraspecific patterns of temperature-size responses and latitude-size clones [21].

## Overview of DRI Analyses

### Currently used methods: their strengths and caveats

Five statistical methods have been used in DRI analyses of individual species data [10]. They either examine the relationship between developmental stage and LDT temperature or ask whether the proportions of time spent in each developmental stage depend on temperature. However, each of them might suffer from potential statistical artifacts.

Two previously used methods rely on the fact that DRI holds if and only if the LDTs of all studied developmental stages are equal. One of them (Method 1 in [10]) estimates mean and standard error of LDT values from the presumed linear relationship between temperature *T* and the development rate *r* (time^-1^) in the given stage,

r = a + bT (1)

LDT is estimated from eq (1) by the intercept (LDT = –*a*/*b*) of the regression line of development rate on temperature; [22] gave an approximate formula for the standard error of the LDT estimate, but a rigorous procedure to test for equality among two or more LDT values in different developmental stages is not available [10]. Another method (Method 2 in [10]) therefore multiplies eq (1) by *d*/*b*, where *d* = *r* ^-1^ is the developmental time in a given stage [23]. Rearranging the terms leads to an estimate of the slope of a new linear regression,

dT =SET+LDTd (2)

which links developmental time *d* to the sum of degree-days *d* T. The intercept *SET* = 1/*b* represents the sum of effective temperatures, equal to the number of day-degrees above the LDT required to complete a particular developmental stage. ANCOVA is used to test if the slopes of the relationship, i.e. the LDT values, are equal across all developmental stages [10]. However, the (random) values of *d* introduce a hidden correlation between the explanatory and explained variable when individual-level data are used (see Fig 1 in [10] for an example) and the explanatory variable (*d*) no longer corresponds to a directly manipulated variable, i.e. ANCOVA assumptions are violated [24].

Shi *et al*. [25] developed another method (Method 5 in [10]) based on the rotation of regression lines to test the independence of LDT on temperature with a Chow test [26]. This is sufficient for two regression lines. To compare LDTs of more than two developmental stages with this method, Kuang *et al*. [10] proposed multiple pairwise comparisons of all regression lines. Increased risk of type I error in such analyses, neglected in [10], can be amended with procedures controlling the family-wise error rate (such as Bonferroni or Holm correction) or the false discovery rate, but a full consensus on their use among ecologists seems to be lacking [27].

The most widely used DRI test was introduced in [5]. It uses angular transformation of the proportional data ($\text{arcsin}\sqrt{p_{i}}$, where *p*_*i*_ is the relative time spent in stage *i*). Linear regression of these transformed data against temperature (Method 3 in [10]) or ANOVA (Method 4 in [10]) is used to assess if relative developmental time of each stage changes with temperature. Factors such as sex, photoperiod, food quality or geographic origin can be included as additional explanatory variables in the linear regression or an ANCOVA analysis [5]. This method is usually applied to mean values of *p*_*i*_ reported in experiments because many papers, especially older ones, do not report individual-level data. Kuang *et al*. [10] suggested that individual-level values of *p*_*i*_ can be transformed and assessed in a similar way as the mean values. This approach would, however, leave out the individual identity, i.e. the fact that the sum of *p*_*i*,*j*_ equals 1 for each individual *j*.

However, the use of angular transformation in the analysis of ecological data is now considered outdated. In addition to the general objections raised in [28], Methods 3 and 4 often suffer from another previously unreported flaw. The values of *p* usually fall in the (0.2, 0.8) range, in which $\text{arcsin}\sqrt{p}$ is nearly linear ($\text{arcsin}\sqrt{p}\approx p+0.2854$) and, as the sum of all relative durations of *k* developmental stages equals 1, the sum of transformed proportions remains close to 1+0.285 *k*. This issue is particularly relevant for datasets with 2–4 developmental stages of which none dominates in duration. Such transformed data then do not pass the assumption of independent data required for linear regression, ANOVA and ANCOVA. Moreover, results in [5] have been inadvertently loaded with two additional issues that might introduce bias both in favour of or against DRI (S1 Text). The net result of these biases cannot be assessed without a detailed reanalysis of all published data on DRI, which is beyond the scope of this study.

Last but not least, Method 3 used for population mean values in is not suitable for data obtained at two temperatures. Most older studies commonly reported only mean developmental times of each stage at each temperature (i.e. *n* = 1 for each developmental stage and temperature). It is not possible to assess DRI from such data at two temperatures because linear regression and ANOVA yield perfect fit unless there are replicated measurements across another factor (see [5] for examples). Datasets with two temperatures have therefore been out of reach for DRI studies. However, if individual-level data are available and there is compelling evidence that the two experimental temperatures lie within the range in which the development rate scales linearly with temperature, there is no reason to abandon an appropriate method such as Dirichlet regression. Test of DRI then simply evaluates if a Dirichlet distribution with temperature-independent parameters fits data measured at the two temperatures.

The above comparison leads us to the proposition that Dirichlet regression is currently the most appropriate and versatile method to analyse DRI data. Its main limitation is the need of individually resolved data, unavailable for many older studies. However, the availability of underlying ecological data has improved over the past decade owing to more widespread data sharing and appearance of new sharing infrastructure and tools [29], making it more and more possible and desirable to replace the standard publication of summary data and statistics by more detailed, individual-level data in new studies. The method developed here does not *a priori* require large datasets and can be used to simultaneously compare models based on various assumptions concerning the effects of temperature and other factors on individual ontogeny. Using data on nine individually reared insect species as a case study, we show that some of the previous conclusions may have been burdened by statistical artifacts. This suggests that the entire concept of DRI should be critically re-evaluated.

### Case study: Dirichlet regression

We apply Dirichlet regression to five species of aquatic and semiaquatic insects (*Cloeon dipterum* (Linnaeus, 1761), *Microvelia reticulata* (Burmeister, 1835), *Velia caprai* (Tamanini, 1947), *Notonecta glauca* (Linnaeus, 1758), and *Acilius canaliculatus* (Nicolai, 1822)) and four species of terrestrial insects (*Amara communis* (Panzer, 1797), *Gastrophysa viridula* (De Geer, 1775), *Leptinotarsa decemlineata* Say, 1824, and *Loxostege sticticalis* (Linnaeus, 1761)) for which we had individually resolved developmental data. We refer to all species by their generic name in the following text and Appendices. Among the (semi)aquatic species, only *Cloeon* is truly aquatic in the sense of relying on dissolved oxygen for respiration; all developmental stages of all other species included in this study breathe atmospheric oxygen. All species were reared individually or in groups in the laboratory at 2–6 different temperatures (S1 Table). Larvae of all species were fed *ad libitum* on a daily basis (see S2 Text for details). No permits were required to collect the individuals in the field and carry out the experiments in agreement with relevant national legislations.

Further details of the experimental protocols varied among species due to their different life histories and environmental requirements. In brief, field-collected adults of terrestrial species were kept in glass or plastic vials and checked for eggs once or twice a day. Overwintered females of *Microvelia* and *Notonecta* laid eggs in the laboratory aquaria, which were randomly placed into one of the experimental temperatures but egg developmental time was not monitored. Attempts to obtain sufficiently large numbers of eggs in the laboratory failed for *Velia*, *Acilius* and *Cloeon*, and early instar larvae collected in the field were instead used in the experiments (see S2 Text for details).

Developmental time (in days) of all larval instars was recorded for *Microvelia* (L1–L5, i.e., all feeding stages); data on L2–L5 for *Velia* and *Notonecta* (i.e., a subset of feeding stages), and data on L2, L3 and pupa for *Acilius*, in which L1–L3 are the feeding stages and pupa is non-feeding. Because *Cloeon* like other mayflies does not have a fixed number of preimaginal developmental stages, we divided its development into a stage containing 1–5 instars from the start of the experiment to the moult into the pre-final instar (hereafter called “early stage”), the pre-final instar, and the final instar before the subimago emerged (see S2 Text for our reasoning supporting this decision); all these stages are feeding. In experiments on terrestrial species, individuals were checked daily or twice a day depending on the species and experimental temperature. Only the hatching of larvae from eggs, pupation and emergence of adults were monitored. We thus collected data on the duration of the egg, larval and pupal stage; only the larval stage is feeding. Because all our data are individually resolved (see S2 Table for the raw data) and Dirichlet regression is applied directly to proportional stage durations without the need to convert them to stage-specific development rates (as in, e.g. [8]), we do not report summary statistics such as time to 50% moult. Total development rate was calculated as the reciprocal value of the total duration of instars included in the analysis of each dataset.

#### Analyses of DRI based in individual-level data

Relative duration of a given developmental stage, i.e. the time spent in that stage divided by the complete developmental time (in days), was calculated for all individuals and stages included in the respective analysis. For *Gastrophysa*, *Leptinotarsa* and *Loxostege*, we analyzed both individual data and mean stage durations for data aggregated by egg clutches or rearing groups. The latter analysis avoids potential pseudoreplication issues and is more appropriate if growth and molting of individuals that develop together is highly synchronized. This was clearly true for leaf beetle eggs that always hatch in synchrony (D. Kutcherov, unpublished data). Previously developed DRI analyses [5,10,22] required the temperatures to be within a range in which the development rate increases linearly with temperature; their major motivation was to obtain a meaningful LDT value. Although our approach does not require such a restriction, we followed those analyses for the sake of direct comparison of the different methods and used linear regression (details not shown) to constrain the data to temperatures for which the criterion was met. This requirement excluded 0–3 temperatures from each dataset; final datasets included 2–4 temperatures in each species.

We compared a suite of Dirichlet regressions differing in the choice of predictors. We used the “common” parameterization in the Dirichlet regression, i.e. we modelled all parameters *α* = (*α*_1_, …, *α*_n_) independently [14], and always included the same set of predictors for all parameters. The simplest model (referred to as *const* in Results and Tables 1 and 2) corresponding to DRI assumed that the proportions of time spent in individual instars are constant and independent of other factors. Another model (*f*) assumed that the proportions are affected by an additional factor (sex: *Acilius*, *Amara*, *Cloeon*; experimental photoperiod: *Amara*, *Gastrophysa*, *Leptinotarsa*; geographic origin of the population: *Loxostege*) but not by temperature. Model *f* therefore corresponds to DRI that differs between sexes, populations, or depends on photoperiod as a factor affecting the rate of development [5]. For *Amara*, we first investigated a model with both sex and photoperiod as two explanatory factors. Inclusion of sex did not significantly improve the fit, and we thus pooled all individuals in the subsequent analyses (details not shown).

**Table 1:** Summary of ΔAIC_c_ values for Dirichlet regression models for individual species of aquatic and semiaquatic insects.

						ΔAIC_c_							
Species	Stages^1^ (*n*)	*N*_T_	*N*_F_	Factor *f*	Note^2^	*const*	*f*	*t*	*t*_F_	*f+t*	*f+t*_F_	f*t	f*t_F_
*Acilius*	L2, L3, pupa (3)	3	2	sex	all temperatures	81.6	85.7	2.1	**1.1**	**1.1**	**0**	6.9	11.6
*Cloeon*	early, pre-final, final (3)	2	2	sex	all temperatures	49.4	27.6	27.5	-	3.3	-	**0**	-
*Microvelia*	L1–L5 (5)	3	2	sex	19–25°C	13.3	21.6	9.6	**0**	19.0	13.3	-	-
*Microvelia*	L1–L5 (5)	3	2	sex	17–21°C	44.7	53.1	53.1	**0**	63.7	14.4	-	-
*Notonecta*	L1–L5 (5)	3	2	sex	all temperatures	31.4	44.2	7.0	**0**	28.5	32.9	-	-
*Velia*	L2–L5 (4)	3	2	sex	12–19°C	86.4	90.4	27.8	**0**	40.6	14.8	-	-

^1^ Stages, temperatures and additional factors: *n* = number of stages; *N*_T_ = number of temperatures; *N*_F_ = number of additional factors.

^2^ Note: Range of temperatures included in the analyses, based on linearity of the relationship between temperature and development rate. Models including the interaction of the additional factor *f* and temperature (models f*t and/or f*t_F_) are given when both the factor and temperature significantly improve the fit. Values of ΔAIC_c_ ≤ 2 for each species and dataset given in bold. See the main text for model abbreviations.

**Table 2:** Summary of ΔAIC_c_ values for Dirichlet regression models for individual species of terrestrial insects.

						ΔAIC_c_										
species	Stages^1^ (*n*)	*N*_T_	*N*_F_	Factor *f*	Note^2^	*const*	*f*	*t*	*t*_F_	*t*+*t* ^2^	*f+t*	*f+t*+*t* ^2^	*f+t*_F_	f*t	f*(*t*+*t* ^2^)	*f* *t_F_
*Amara*	E, L, P (3)	4	2	PP	all temperatures	347	28.5	228	235	-	**0**	3.8	**0.2**	4.9	-	-
*Gastrophysa*	E, L, P (3)	3	2	PP	I, 18–22°C	469	444	132	94.8	-	79.1	-	37.5	46.4	-	**0**
*Gastrophysa*	E, L, P (3)	3	2	PP	C, 18–22°C	23.6	24.6	**1.1**	5.6	-	**0**	-	4.1	**0.9**	-	12.3
*Leptinotarsa*	E, L, P (3)	4	3	PP	I, all temperatures	655	392	394	311	317	84.3	9.4	2.6	91.8	-	**0**
*Leptinotarsa*	E, L, P (3)	3	3	PP	I, 21–27°C	541	298	307	296	-	17.4	-	**0**	15	-	**2.0**
*Leptinotarsa*	E, L, P (3)	4	3	PP	C, all temperatures	168	101	94.7	78.3	78.9	13.4	**0**	**1.5**	25.6	24.3	28.1
*Leptinotarsa*	E, L, P (3)	3	3	PP	C, 21–27°C	123	68.2	67.7	61.7	-	4.9	-	**0**	12.6	-	13.9
*Loxostege*	E, L, P (3)	3	3	O	I, 18–24°C	215	53.6	205	205	-	33.9	-	29.3	33.4	-	**0**
*Loxostege*	E, L, P (3)	3	3	O	C, 21–27°C	15.4	**0**	13.5	20.3	-	**0.3**	-	9.9	6.1	-	66.3

^1^ Stages and additional factors. Stage abbreviations: E = egg, L = larva, P = pupa; factors: PP = photoperiod, O = geographic origin of population.

^2^ Range of temperatures included in the analyses, based on linearity of the relationship between temperature and development rate; I = individual-based data, C = mean clutch values.

Other details as in Table 1.

The remaining models included the effect of temperature. The simplest one (*t*) assumed a linear effect of temperature on the parameters *α* of the underlying Dirichlet distribution. This translates into a nearly, but not perfectly, linear relationship between temperature and relative duration of a given developmental stage. We thus calculated the amount of DRI violation *V*_D_ as the average difference between relative developmental times $\hat{p}$ predicted for two successive temperatures and divided by the temperature difference,

$$V_{\text{D}}=\frac{1}{N_{T}-1}\sum_{i=1}^{N_{T}-1}\frac{\hat{p}\left(T_{i+1}\right)-{\hat{p}}_{i}\left(T_{i}\right)}{T_{i+1}-T_{i}}$$ (3)

and compared it to the measure of DRI violation *V*_A_ introduced in [5]; see eqn (A2) in S1 Text. We also investigated the possibility of independent effects of each temperature (*t*_*F*_), i.e. we treated temperature as a factor for data with three or more different temperatures, and the possibility of a unimodal dependence on temperature described by a second order polynomial (model *t+t* ^2^) for data with four different temperatures. Finally, we included models that consider joint additive effect of the additional factor and temperature (continuous temperature: *f+t* and *f+t+t* ^2^ temperature as factor: *f+t*_F_) or their interaction on model parameters (continuous temperature: f*t and f*(*t+t* ^2^), temperature as factor: f*t_F_). The flexibility of Dirichlet regression models would allow for mutually independent effects of temperature or additional factors on individual model parameters; see [14] for details. We did not explore this possibility as we had no *a priori* hypotheses on which we could base such models. Instead, we assumed that temperature or the additional factor affected all model parameters equally.

For each dataset, we compared all models using Akaike information criterion with correction for small sample sizes (AIC_c_). We chose the model with the lowest AIC_c_ value as the most appropriate description of the underlying relationship; models for which the difference of the AIC_c_ value from the lowest value is at most 2 and hence their evidence ratio does not deviate too strongly from unity also provide good fit to the data [30]. Comparing the AIC_c_ values of models *const* and *t*_F_ (or AIC_c_ values of model *f* and *f+t*_F_) is analogous to the deletion tests used in [5] to determine if the data are consistent with DRI.

#### Comparison of Dirichlet regression and standard ANCOVA analyses of DRI

To illustrate the differences between Dirichlet regression and previously used methods, we also carried out the ANCOVA analysis of mean values of the transformed proportional data following the procedure outlined in [5], i.e. Method 3 in [10]. We used the same datasets as in Dirichlet regression and compared the measures of DRI violation *V*_D_ and *V*_A_ across species. We also examined the dependence of DRI violation measure *V*_D_ on ontogeny. To do so, we rescaled individual stages of pre-adult ontogeny of each species between 0 and 1 using equidistant intervals with the resolution depending on that of the data: we use 0 for egg, 0.5 for the combined larval stages and 1 for pupa of the four terrestrial species, the values of 0.25, 0.5, 0.75 and 1 for L1–L3 larvae and pupa of *Acilius* and the values of 0.2, 0.4, 0.6, 0.8 and 1 for L1–L5 larvae of Heteroptera. Finally, we assigned the values of 0.5, 0.75 and 1 to early, pre-final and final instars of *Cloeon*. These values are somewhat arbitrary but different values did not qualitatively change our conclusions (results not shown). More than one dataset could be analyzed for some species (either the linear dependence of development rate on temperature could be achieved for different, partly non-overlapping temperature intervals, or we wanted to highlight differences between individual- and clutch-based data). To avoid pseudoreplication, we averaged the values of DRI violation resulting from these analyses for each stage of each species across all datasets and levels of a factor.

All analyses were implemented in R software version 3.1.0 [31]; Dirichlet regression was implemented in the DirichletReg package v 0.6–0 [14] and graphs drawn in the ggplot2 package [32]. An example of the Dirichlet regression analysis is provided in S3 Text.

## Results

DRI was violated in all species based on the results from Dirichlet regression except *Loxostege*, in which the best model was consistent with DRI for the clutch-aggregated data but suggested DRI violation for the individual-level data (Tables 1 and 2). Models including temperature as a factor (*t*_*F*_) were clearly favoured in the sense of having the lowest AIC_c_ scores in three species: *Velia*, *Microvelia* and *Notonecta*. We found no support for sex-specific development in these species (Table 1).

**Fig 2:**
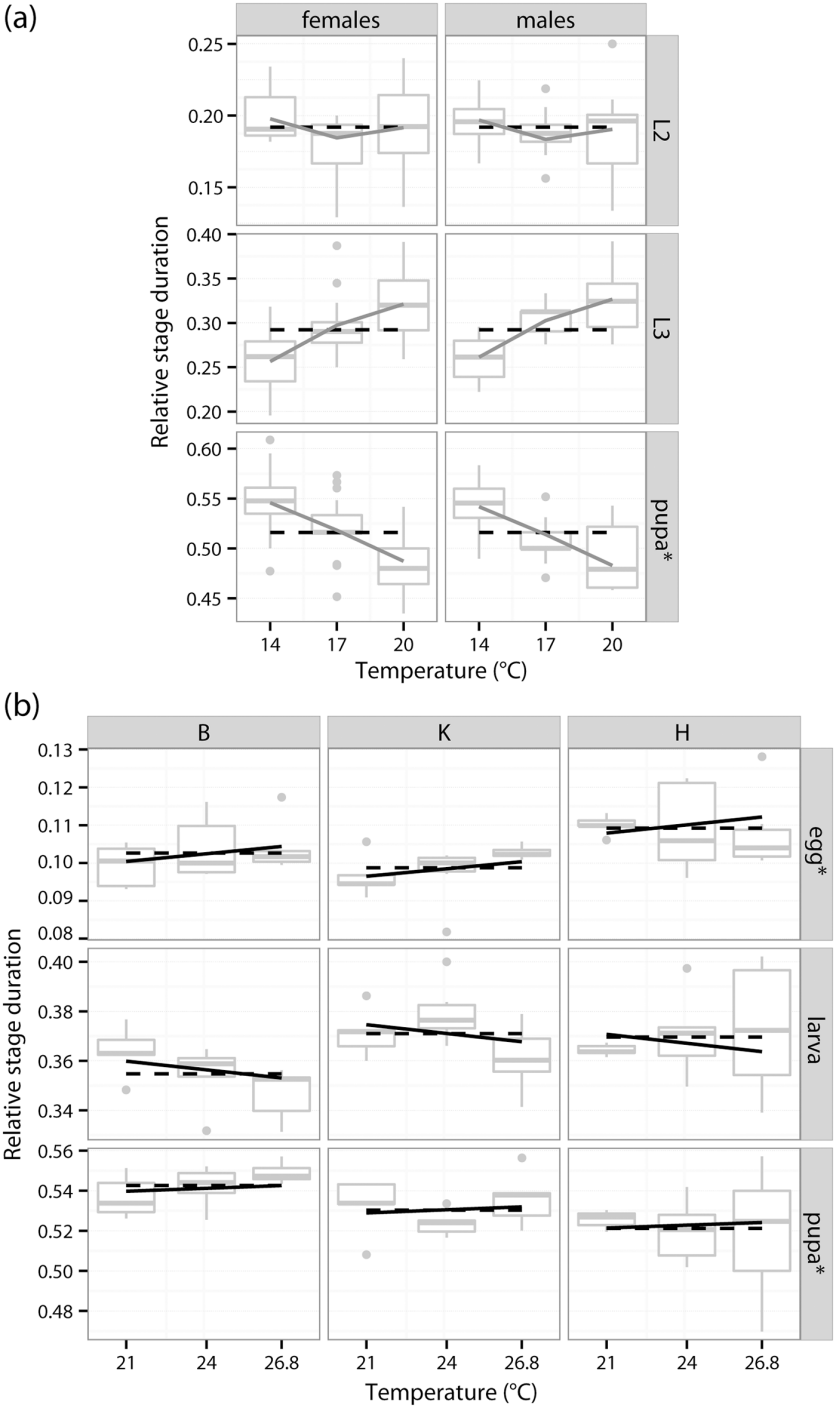
Examples of results of Dirichlet regression for (a) *Acilius* and (b) *Loxostege*. *Acilius*: best fitting model (*f+t*_F_, grey solid line) compared to DRI (model *const*, black dashed lines); *Loxostege*: dataset with average clutch data, 21–27°C and different population origin (B = Buryatia, K = Krasnodar, H = Hebei), best fitting DRI model (*f*, black dashed lines) compared to model *f+t* (black solid lines). Box and whisker plots of raw data: horizontal line = median, box = first to third quartiles, line = data within 1.5 times the interquartile range; dots = outliers. Non-feeding stages labelled with asterisk.

Inclusion of an additional factor, sometimes in interaction with temperature, led to a significantly improved model in all remaining species and datasets: sex in *Cloeon* and *Acilius* (although the differences between male and female *Acilius* were minor), photoperiod in *Amara*, *Gastrophysa* and *Leptinotarsa*, and geographic origin in *Loxostege* (Fig 2 and Tables 1 and 2). Other model variants involving the factor and temperature usually provided a similarly good fit of the data (bold values in Tables 1 and 2) and their predictions were similar to the best-fitting model (details not shown).

**Fig 3:**
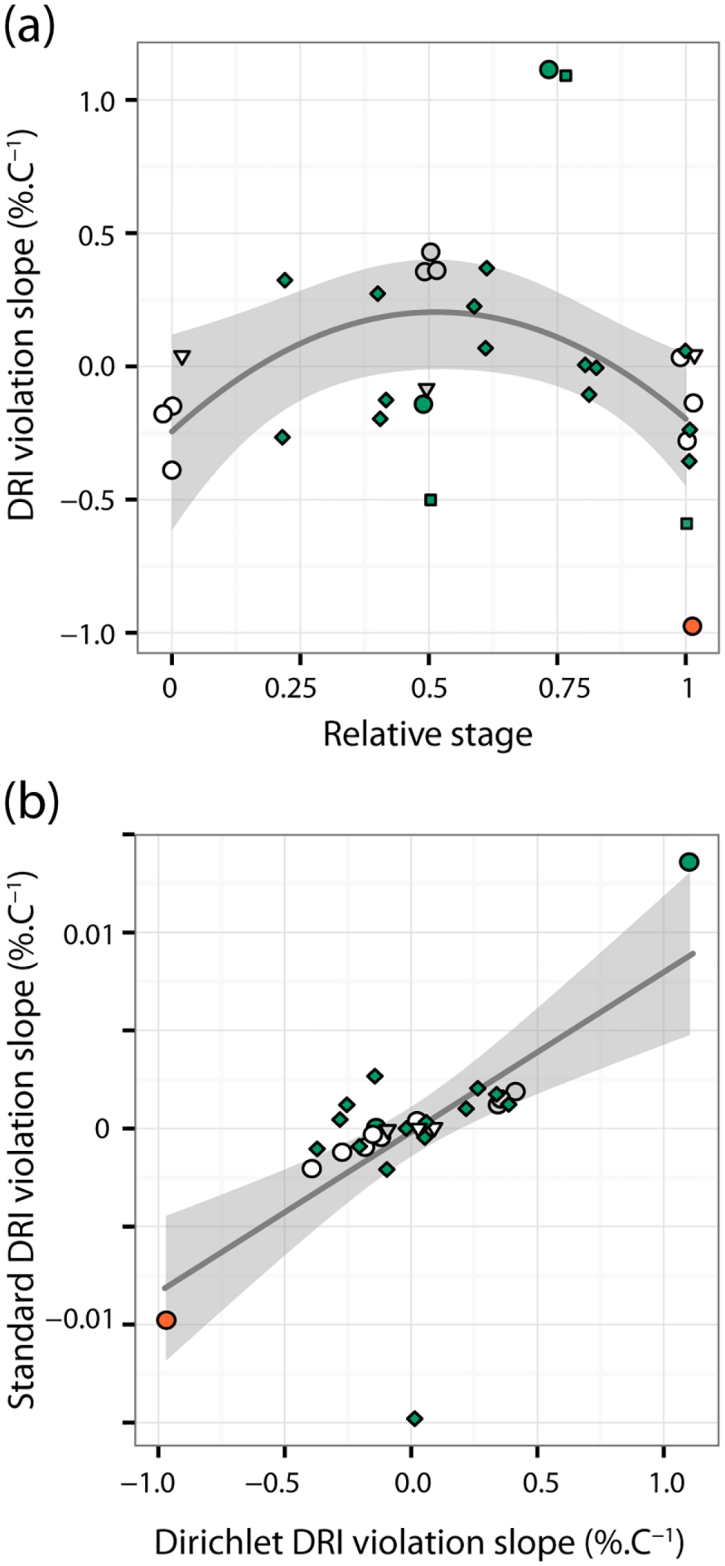
DRI violation in the nine insect species studied. (a) Average stage-specific DRI violation from Dirichlet regression. Stages on a relative scale (0 = egg, 1 = last pre-adult stage). Curve ± 95% confidence interval = best model describing the dependence of DRI violation *V*_D_ on the relative developmental stage *S* (see main text for details). (b) Comparison of average stage-specific DRI violation from Dirichlet regression and standard ANCOVA analysis as described in Jarošík et al. (2002). Line ± 95% confidence interval = regression of *V*_A_ on *V*_D_ (see main text for details). Symbols represent values averaged across all levels of a factor and all datasets for each species included in Tables 1 and 2; small amount of horizontal jitter added to all data. Green fill = feeding stages of aquatic species, orange fill = non-feeding stages of aquatic species, grey fill = feeding stages of terrestrial species, no fill = non-feeding stages of terrestrial species; circles = Coleoptera, squares = *Cloeon*, diamond = Heteroptera, triangles = *Loxostege*.

Although DRI violation detected by Dirichlet regression varied among the species, we observed a common pattern suggesting that the relative duration of intermediate developmental stages increased with temperature at the expense of shortened early instars and the last pre-adult instars (Fig 3a). This pattern did not differ significantly between aquatic and terrestrial species. The best model describing the DRI violation detected by Dirichlet regression *V*_D_ as a function of relative developmental stage *S* and habitat included only a quadratic dependence on *S*, *V*_D_ = -0.24 + 1.73 *S–* 1.69 *S* ^2^ (*F*_2,29_ = 3.61, P = 0.040, adj. *r* ^2^ = 0.14), and the linear and quadratic coefficients were both significantly different from zero (*P* = 0.02 and 0.01, respectively). DRI violation was strongest in *Cloeon* and *Acilius*, in which the relative development rate of the intermediate stages included in the experiment (pre-final instar in *Cloeon* and L3 in *Acilius*) increased by more than 1%.(°C)^-1^. Removing these two species did not qualitatively change the results but increased the proportion of explained variance (*V*_D_ = -0.15 + 1.38 *S–* 1.38 *S* ^2^ (*F*_2,18_ = 6.12, P = 0.009, adj. *r* ^2^ = 0.34). The averaged slope of DRI violation did not exceed 0.5%.(°C)^-1^ in all other species and stages (Fig 3a).

ANCOVA analysis of mean values of the transformed proportional data following the procedure outlined in [5] revealed DRI violation in only four species (*Amara*, *Gastrophysa*, *Leptinotarsa* and *Acilius*). Moreover, it did not find a significant effect of the additional factor on relative developmental times in *Acilius* and *Gastrophysa* detected by Dirichlet regression (S3 Table). Finally, the best model describing the back-transformed values of DRI violation *V*_A_ detected by the ANCOVA analysis as a function of relative developmental stage *S* and habitat included only the intercept, *V*_A_ = -1.7·10^−4^; the result was qualitatively identical when we dropped data for *Acilius* that had the highest leverage. This means that the standard DRI analysis could not detect variation in the temperature dependence of development rates during ontogeny. Magnitude of *V*_A_ did not exceed 0.15%.(°C)^-1^ and was typically two orders of magnitude lower than *V*_D_ (linear regression model: *V*_A_ = -0.00019 +0.0082 *V*_D;_ *F*_1,27_ = 22.4, P < 10^−4^, adj. *r* ^2^ = 0.43; Fig 3b) for reasons explained in S1 Text.

## Discussion

Developmental rate isomorphy, akin to equiproportional development, is an important life history concept. Its widespread validity would greatly simplify efforts to understand the responses of individuals and populations to the anticipated climate change, because it implies that species respond uniformly to changing temperature during ontogeny [5]. Moreover, the ontogeny of species that comply with DRI could be conveniently characterized by only two numbers, the lower developmental threshold (LDT) below which the development should stop, and the slope relating development rate to temperature [5]. Previous studies found that DRI holds in many ectotherms and that violations of DRI are minor [5,11]. On the other hand, studies on EPD indicate that the concept is often violated in copepods [6,8].

Our results suggest that universal validity of DRI is unlikely. Most importantly, multiple characteristics of individuals that may alter the rates of growth and development change during ontogeny (e.g., ontogenetic diet and niche shifts [33,34]). Recent meta-analyses and experiments show that the upper thermal limit of growth of different species decreases with their body size [19,20]. If the underlying interspecific allometries also apply within some species, DRI will not hold for them unless the changes in growth rates are perfectly matched by changes in development rates. In our case study, DRI was violated in at least eight of the nine species when we used individual-level data. Contrary to our results, previous analyses of data on *Leptinotarsa* and *Gastrophysa* mostly supported DRI (*Leptinotarsa*: [5]; *Gastrophysa*: [35], but see [36]).

Results of our case study point towards three limitations of previous DRI studies. First, the different results may arise from differences in statistical methods and data resolution explained above. Second, most previous studies have grouped all larval instars together. We show that using individual instars could detect DRI violation at higher resolution. Third, previous coverage of taxa might have been naturally biased. Although Jarošík *et al*. [11] extended the coverage of DRI to other invertebrate and vertebrate ectotherms (echinoderms, annelids, fish and anurans), the main bulk of DRI evidence remains rooted in insect studies. A large proportion of data come from economically important terrestrial species, usually pests and disease vectors and their predators; see [5]. Data on aquatic and semiaquatic insects are scarce: they are usually of low economic importance and do not include typical model species used for laboratory studies. On the other hand, different thermal conditions and temperature-dependent aerobic scopes in terrestrial and aquatic environments may lead to environment-specific patterns of growth and development [37,38] and ultimately to different temperature-size relationships [21].

Neither is the current DRI concept fully suitable to characterize species with non-linear dependence of development rate on temperature [8] and populations in suboptimal habitats, in which variable food limitation over ontogeny may further alter temperature dependence of development rates [7]. Future studies of DRI should therefore include both terrestrial and (semi)aquatic species, relax the constraint on linear relationship between development rate and temperature, and consider limiting food conditions. To this end, joint analyses of data from EPD and DRI studies would be particularly useful.

### Violation of DRI: Possible causes and consequences

To identify which environmental factors and life history traits are responsible for the violation of DRI, individually resolved data on different generations in multivoltine populations and comparisons of related species with different voltinism or inhabiting environments with different thermal amplitudes would be particularly useful. We found no differences between the overall pattern of DRI violation in terrestrial and aquatic insects included in our study. This is unexpected, given that aquatic insects should experience smaller temperature fluctuations due to the higher thermal capacity of water and might perceive the environment as more predictable than terrestrial insects. We could not test if different aerobic scope in aquatic and terrestrial environments drives the patterns of DRI violations because our study included only one truly aquatic species (*Cloeon*) that does not breathe atmospheric oxygen.

The main pattern of DRI violation found in our analyses was shared among species: relative duration of the early and the last preimaginal stages decreased with temperature, whereas intermediate instars tended to last relatively longer. This result is consistent with our second hypothesis based on the narrowing metabolic scope of growth at higher temperatures, which affects the actively foraging larval stage but not the eggs or pupae (Fig 1d). This explanation is not satisfactory only for the Heteroptera, in which even the last larval instar actively forages for food. However, the patterns of DRI violation in the Heteroptera were generally variable (Fig 3a) and other currently unknown factors and mechanisms may be responsible for the result.

Seasonal constraints on development and variation in photoperiod can also lead to DRI violation. For example, absolute durations of egg and pupal stage were not affected by photoperiod in our data on *Amara* and *Leptinotarsa*, but the rate of larval development changed with day length and consequently changed the relative duration of all three stages. Other published data (e.g. for the damselfly *Lestes eurinus*, [39]) also suggest DRI violation under late-season conditions, presumably as some late-instar individuals accelerate growth to emerge before the end of the season and to avoid overwintering. The resulting pattern is qualitatively identical with our findings; its proximate cause includes a joint effect of temperature and day length on development rates (e.g. [40]).

## Conclusions

We conclude that the DRI and EPD concepts developed for different taxa should be unified and possible patterns and causes of DRI/EPD violation critically re-evaluated. As we have illustrated, a fruitful approach could utilize the concept of stage- or size-specific thermal performance curves. Contrary to most previous studies, our experiments indicated that DRI is often violated in ectotherms and that this violation can be substantial. Using individually resolved data, we found DRI violation in insects that are both terrestrial and (semi)aquatic, predatory and herbivorous, and hemi- and holometabolous. We therefore suggest that modern statistical methods applied to individual-level data should be employed in DRI analyses whenever possible. Dirichlet regression used in this paper provides a highly flexible instrument that explicitly deals with the constraints imposed by proportional data that inherently arise in the study of DRI/EPD.

## Supporting Information

S1 Table Summary of experimental data used in the analyses. (DOC)

S2 Table Raw individual-level data on all nine species used in the analyses. (XLS)

S3 Table Results of standard DRI analysis. (DOC)

S1 Text Properties of the measure of DRI violation proposed in Jarošík et al. (2002). (DOC)

S2 Text Study organisms and experimental details. (DOC)

S3 Text Example of DRI analysis using Dirichlet regression. (DOC)

## References

[1] Huey R, Kingsolver J. Variation in universal temperature dependence of biological rates. Proc Natl Acad Sci U S A. 2011;108: 10377–10378. 10.1073/pnas.1107430108 21680885 PMC3127921

[2] Corkett CJ, McLaren IA. Relationships between development rate of eggs and older stages of copepods. J Mar Biol Assoc United Kingdom. 1970;50: 161–168.

[3] Corkett CJ. Observations on development in copepods. Crustaceana. 1984;(Suppl.) 7: 150–153.

[4] Van Rijn PCJ, Mollema C, Steenhuis-Broers GM. Comparative life history studies of Frankliniella occidentalis and Thrips tabaci (Thysanoptera: Thripidae) on cucumber. Bull Entomol Res. 1995;85: 285–297.

[5] Jarošík V, Honěk A, Dixon A. Developmental rate isomorphy in insects and mites. Am Nat. 2002;160: 497–210. 10.1086/342077 18707525

[6] Hart RC. Copepod post-embryonic durations: pattern, conformity, and predictability. The realities of isochronal and equiproportional development, and trends in the opepodid-naupliar duration ratio. Hydrobiologia. 1990;206: 175–206.

[7] Hart RC. Copepod equiproportional development: Experimental confirmation of its independence of food supply level, and a conceptual model accounting for apparent exceptions. Hydrobiologia. 1998;380: 77–85.

[8] Forster J, Hirst AG, Woodward G. Growth and development rates have different thermal responses. Am Nat. 2011;178: 668–678. 10.1086/662174 22030735

[9] Perdikis DC, Fantinou AA, Lykouressis DP. Constant rate allocation in nymphal development in species of Hemiptera. Physiol Entomol. 2003;28: 331–339.

[10] Kuang X-J, Parajulee MN, Shi P-J, Ge F, Xue F-S. Testing the rate isomorphy hypothesis using five statistical methods. Insect Sci. 2012;19: 121–128.

[11] Jarošík V, Kratochvíl L, Honěk A, Dixon AFG. A general rule for the dependence of developmental rate on temperature in ectothermic animals. Proc R Soc B Biol Sci. 2004;271 Suppl: S219–21. 15252989 10.1098/rsbl.2003.0145 PMC1810016

[12] Dell AI, Pawar S, Savage VM. Temperature dependence of trophic interactions are driven by asymmetry of species responses and foraging strategy. J Anim Ecol. 2014;83: 70–84. 10.1111/1365-2656.12081 23692182

[13] Aitchison J. The Statistical Analysis of Compositional Data. Caldwell, USA: The Blackburn Press; 2003.

[14] Maier MJ. DirichletReg: Dirichlet Regression for Compositional Data in R. 2014. Report No.: 125.

[15] Kotz S, Johnson NL, Balakrishnan N. Continuous multivariate distributions. 2nd ed New York, USA: Wiley-Blackwell; 2000.

[16] Nylin S, Gotthard K. Plasticity in life-history traits. Annu Rev Entomol. 1998;43: 63–83. 9444750 10.1146/annurev.ento.43.1.63

[17] Trudgill DL. Why do tropical poikilothermic organisms tend to have higher threshold temperatures for development than temperate ones. Funct Ecol. 1995;9: 136–137.

[18] Stearns SC. The evolutionary significance of phenotypic plasticity. Bioscience. 1989;39: 436–445.

[19] Vucic-Pestic O, Ehnes RB, Rall BC, Brose U. Warming up the system: higher predator feeding rates but lower energetic efficiencies. Glob Chang Biol. 2011;17: 1301–1310.

[20] Fussmann KE, Schwarzmüller F, Brose U, Jousset A, Rall BC. Ecological stability in response to warming. Nat Clim Chang. 2014;4: 206–210.

[21] Horne CR, Hirst AG, Atkinson D. Temperature-size responses match latitudinal-size clines in arthropods, revealing critical differences between aquatic and terrestrial species. Ecol Lett. 2015;18: 327–335. 10.1111/ele.12413 25682961

[22] Campbell A, Frazer BD, Gilbert N, Gutierrez AP, Mackauer M. Temperature requirements of some aphids and their parasites. J Appl Ecol. 1974;11: 431–438.

[23] Ikemoto T, Takai K. A new linearized formula for the law of total effective temperature and the evaluation of line-fitting methods with both variables subject to error. Environ Entomol. 2000;29: 671–682.

[24] Sokal RR, Rohlf FJ. Biometry: the principles and practice of statistics in biological research. 3rd ed New York, USA: W. H. Freeman; 1995.

[25] Shi P, Ge F, Men X. How to compare the lower developmental thresholds. Environ Entomol. 2010;39: 2033–2038. 10.1603/EN10136 22182571

[26] Chow GC. Tests of equality between sets of coefficients in two linear regressions. Econometrica. 1960;28: 591–605.

[27] García L. Escaping the Bonferroni iron claw in ecological studies. Oikos. 2004;105: 657–663.

[28] Warton DI, Hui FKC. The arcsine is asinine: the analysis of proportions in ecology. Ecology. 2011;92: 3–10. 21560670 10.1890/10-0340.1

[29] Pham-Kanter G, Zinner DE, Campbell EG. Codifying collegiality: recent developments in data sharing policy in the life sciences. PLoS One. 2014;9: e108451 10.1371/journal.pone.0108451 25259842 PMC4178158

[30] Burnham KP, Anderson DR. Model selection and multimodel inference: a practical information-theoretic approach. 2nd ed New York, USA: Springer-Verlag; 2002.

[31] R Core Team. R: A language and environment for statistical computing. Vienna, Austria: R Foundation for Statistical Computing; 2014 10.1016/j.jneumeth.2014.06.019

[32] Wickham H. Ggplot2: elegant graphics for data analysis. New York, USA: Springer; 2009.

[33] Miller TEX, Rudolf VHW. Thinking inside the box: community-level consequences of stage-structured populations. Trends Ecol Evol. 2011;26: 457–466. 10.1016/j.tree.2011.05.005 21680049

[34] Klecka J, Boukal DS. Who eats whom in a pool? A comparative study of prey selectivity by predatory aquatic insects. PLoS One. 2012;7: e37741 10.1371/journal.pone.0037741 22679487 PMC3367957

[35] Honěk A, Jarošík V, Martínková Z. Effect of temperature on development and reproduction in Gastrophysa viridula (Coleoptera: Chrysomelidae). Eur J Entomol. 2003;100: 295–300.

[36] Kucherov DA, Kipyatkov VE. Control of preimaginal development by photoperiod and temperature in the dock leaf beetle Gastrophysa viridula (De Geer) (Coleoptera, Chrysomelidae). Entomol Rev. 2011;91: 692–708.

[37] Ward JV, Stanford J. Thermal responses in the evolutionary ecology of aquatic insects. Annu Rev Entomol. 1982;27: 97–117.

[38] Atkinson D. Effects of temperature on the size of aquatic ectotherms: exceptions to the general rule. J Therm Biol. 1995;20: 61–74.

[39] Lutz P. Effects of temperature and photoperiod on larval development in Lestes eurinus (Odonata: Lestidae). Ecology. 1968;49: 637–644.

[40] Kutcherov DA, Lopatina EB, Kipyatkov VE. Photoperiod modifies thermal reaction norms for growth and development in the red poplar leaf beetle Chrysomela populi (Coleoptera: Chrysomelidae). J Insect Physiol. Elsevier Ltd; 2011;57: 892–898. 10.1016/j.jinsphys.2011.03.028 21510952

