# Supplementary material for: Analyses of Developmental Rate Isomorphy in Ectotherms: Introducing the Dirichlet Regression
Source: PLoS One. 2015 Jun 26;10(6):e0129341. doi: 10.1371/journal.pone.0129341 (PMC4482627; doi:10.1371/journal.pone.0129341)
Supplement: S1 Table — (DOC) [file pone.0129341.s001.doc]

**S2 Table.** **Summary of experimental data used in the analyses.** Factor (excluding sex): PP = photoperiod, O = geographic origin of population. *n*_i_ = initial number of individuals, both sexes pooled (number of egg batches or groups in parentheses), *n* = number of surviving individuals, both sexes pooled (number of surviving clutches or groups in parentheses). Asterisk = data from an earlier experiment (Ditrich T, Papáček M. Eur J Entomol. 2010;107: 579–587).

| Species | Temperature (°C) | Factor | *n_i_* | *n* |
| --- | --- | --- | --- | --- |
| *Acilius* | 14 | - | 51 | 37 |
|  | 17 | - | 50 | 33 |
|  | 20 | - | 52 | 35 |
| *Cloeon* | 14.3 | - | 48 | 43 |
|  | 18.3 | - | 48 | 38 |
| *Microvelia* | 17 | - | 20 | 12 |
|  | 19* | - | 48* | 45* |
|  | 21 | - | 20 | 20 |
|  | 25 | - | 20 | 18 |
| *Notonecta* | 17 | - | 20 | 10 |
|  | 21 | - | 20 | 14 |
|  | 25 | - | 20 | 16 |
| *Velia* | 12 | - | 40 | 25 |
|  | 15 | - | 40 | 16/25 |
|  | 19* | - | 48* | 6*/12* |
|  | 23 | - | 40 | 8/17 |
| *Amara* | 16 | PP (12L:12D) | 110 | 30 |
|  | 18 | PP (12L:12D) | 110 | 41 |
|  | 20 | PP (12L:12D) | 112 | 45 |
|  | 22 | PP (12L:12D) | 111 | 37 |
|  | 16 | PP (22L:2D) | 111 | 11 |
|  | 18 | PP (22L:2D) | 110 | 16 |
|  | 20 | PP (22L:2D) | 110 | 31 |
|  | 22 | PP (22L:2D) | 110 | 22 |
| *Gastrophysa* | 16 | PP (12L:12D) | 657 (18) | 368 (18) |
|  | 18 | PP (12L:12D) | 702 (19) | 417 (18) |
|  | 20 | PP (12L:12D) | 736 (20) | 439 (20) |
|  | 22 | PP (12L:12D) | 716 (19) | 287 (19) |
|  | 23.8 | PP (12L:12D) | 775 (20) | 256 (20) |
|  | 25.7 | PP (12L:12D) | 628 (20) | 223 (20) |
|  | 16 | PP (22L:2D) | 720 (21) | 417 (21) |
|  | 18 | PP (22L:2D) | 681 (20) | 392 (20) |
|  | 20 | PP (22L:2D) | 621 (19) | 327 (19) |
|  | 22 | PP (22L:2D) | 692 (19) | 223 (19) |
|  | 23.8 | PP (22L:2D) | 672 (20) | 221 (20) |
|  | 25.7 | PP (22L:2D) | 659 (20) | 164 (20) |
| *Leptinotarsa* | 18 | PP (12L:12D) | 290 (38) | 36 (18) |
|  | 21 | PP (12L:12D) | 226 (36) | 81 (24) |
|  | 24 | PP (12L:12D) | 392 (41) | 94 (27) |
|  | 27 | PP (12L:12D) | 253 (40) | 109 (30) |
|  | 18 | PP (18L:6D) | 302 (39) | 45 (18) |
|  | 21 | PP (18L:6D) | 337 (36) | 82 (22) |
|  | 24 | PP (18L:6D) | 281 (35) | 89 (23) |
|  | 27 | PP (18L:6D) | 333 (39) | 138 (31) |
|  | 18 | PP (22L:2D) | 296 (42) | 38 (19) |
|  | 21 | PP (22L:2D) | 300 (40) | 71 (22) |
|  | 24 | PP (22L:2D) | 372 (37) | 94 (22) |
|  | 27 | PP (22L:2D) | 302 (41) | 109 (35) |
| *Loxostege* | 18 | O (Buryatia) | 265 (6) | 84 (6) |
|  | 21 | O (Buryatia) | 247 (5) | 105 (5) |
|  | 24 | O (Buryatia) | 224 (5) | 44 (5) |
|  | 26.8 | O (Buryatia) | 303 (5) | 132 (5) |
|  | 18 | O (Krasnodar) | 273 (5) | 69 (5) |
|  | 21 | O (Krasnodar) | 249 (5) | 108 (5) |
|  | 24 | O (Krasnodar) | 246 (6) | 59 (6) |
|  | 26.8 | O (Krasnodar) | 307 (5) | 65 (5) |
|  | 18 | O (Hebei) | 162 (5) | 41 (5) |
|  | 21 | O (Hebei) | 271 (5) | 87 (5) |
|  | 24 | O (Hebei) | 217 (5) | 47 (5) |
|  | 26.8 | O (Hebei) | 245 (6) | 67 (6) |
