# Supplementary material for: Analyses of Developmental Rate Isomorphy in Ectotherms: Introducing the Dirichlet Regression
Source: PLoS One. 2015 Jun 26;10(6):e0129341. doi: 10.1371/journal.pone.0129341 (PMC4482627; doi:10.1371/journal.pone.0129341)
Supplement: S3 Table — (DOC) [file pone.0129341.s003.doc]

**S6 Table. Results of standard DRI analysis.** The analysis followed the ANCOVA procedure as outlined in Jarošík *et al.* (2002). We analyzed the same data as specified in the main text in Table 1 and 2 except *Cloeon*, for which only data at two temperatures were available and the analysis could not be performed. The table summarizes the following statistically significant results for the factor: interaction between the factor and stage (column ‘*f ** stage’) and factor-dependent slope and intercept of the regression of the relative developmental time on temperature. *P* gives the probability that the regression slope(s) are significantly different from zero. Values of *P* < 0.05 indicating a violation of DRI in bold; separate probabilities are reported when each stage was analysed separately. Factor abbreviations and notes as in Tables 1 and 2 in the main text.

|  |  |  | Significant factor effects | | | | | | |
| --- | --- | --- | --- | --- | --- | --- | --- | --- | --- |
| Species | Factor *f* | Note | *f ** stage | slope | intercept | *P* | *P*_egg_ | *P*_larva_ | *P*_pupa_ |
| *Amara* | PP | all temperatures | Yes | No | Yes | - | **0.019** | **0.002** | **0.009** |
| *Gastrophysa* | PP | I, 18‒22°C | No | No | No | **0.038** | - | - | - |
| *Gastrophysa* | PP | C, 18‒22°C | No | No | No | **0.025** | - | - | - |
| *Leptinotarsa* | PP | I, all temperatures | Yes | No | Yes | - | **0.001** | **0.002** | **0.017** |
| *Leptinotarsa* | PP | I, 21‒27°C | Yes | No | Yes | - | **7.310^-5^** | **3.010^-4^** | **0.026** |
| *Leptinotarsa* | PP | C, all temperatures | Yes | No | Yes | - | **0.002** | **0.006** | **0.038** |
| *Leptinotarsa* | PP | C, 21‒27°C | Yes | No | Yes | - | **0.002** | **0.019** | 0.11 |
| *Loxostege* | O | I, 18‒24°C | Yes | No | Yes | - | 0.83 | 0.79 | 0.83 |
| *Loxostege* | O | C, 21‒27°C | Yes | No | Yes | - | 0.16 | 0.38 | 0.56 |
| *Acilius* | sex | all temperatures | No | No | No | **0.021** | - | - | - |
| *Microvelia* | sex | 19–25°C | No | No | No | 0.70 | - | - | - |
| *Microvelia* | sex | 17–21°C | No | No | No | 0.91 | - | - | - |
| *Notonecta* | sex | all temperatures | No | No | No | 0.41 | - | - | - |
| *Velia* | sex | 12–19°C | No | No | No | 0.38 | - | - | - |
