# Supplementary material for: Analyses of Developmental Rate Isomorphy in Ectotherms: Introducing the Dirichlet Regression
Source: PLoS One. 2015 Jun 26;10(6):e0129341. doi: 10.1371/journal.pone.0129341 (PMC4482627; doi:10.1371/journal.pone.0129341)
Supplement: S1 Text — (DOC) [file pone.0129341.s004.doc]

**S1 Text: Properties of the measure of DRI violation proposed in Jarošík et al. (2002).**

Jarošík *et al.* (2002) measured DRI violation *V*_A_ in percents of change in stage duration per degree Celsius by transforming the slope of the resulting slope *z* of the regression

$\text{arcsin}\sqrt{p}=a+z T$ (A1)

back to proportions as

$V_{\text{A}}\text{=100 sin}^{2}\left(z\right)$ (A2)

However, the relationship between *V*_A_ and the slope of the relationship for the untransformed data is not straightforward because transforming (A1) back to raw proportions yields

$p=\text{sin}^{2}\left(a+z T\right)\text{ }$. (A3)

Hence, a more appropriate value of the slope can be found by differentiating (A3) with respect to temperature:

$\tilde{V}_{\text{A}}=100 \frac{\text{d}\left(\text{sin}^{2}\left(a+z T\right)\right)}{\text{d}T}\text{ =}200 z \text{cos}\left(a+z T\right)\text{sin}\left(a+z T\right)$, (A4)

which is different from the formula proposed by (Jarošík *et al.* 2002).

For intermediate values of *p*, the value of *p* + 0.2854 provides a close approximation to $\text{arcsin}\sqrt{p}$, so that (A1) yields $p\approx a+z T-0.2854$ and (A4) simplifies to

$\tilde{V}_{\text{A}}\approx100 \frac{\text{d}\left(a+z T-0.2854\right)}{\text{d}T}\text{ =1}00 z .$ (A5)

As $z \ll1$ and therefore $\text{sin} \text{(}z\text{)}\approx z$ always holds for the DRI data, including those reported in Table A1 in Jarošík *et al.* (2002), the ratio between $\tilde{V}_{\text{A}}$ calculated from (A3) and the value calculated in Jarošík *et al.* (2002) equals $V_{\text{A}}/\tilde{V}_{\text{A}}\approx\text{ }z$. This means that *V*_A_ always underestimates the true slope of the DRI violation, usually by 2–4 orders of magnitude (see Jarošík *et al.* (2002), Table A1, mean values of the slope of the angular transformation).
