# Supplementary material for: Analyses of Developmental Rate Isomorphy in Ectotherms: Introducing the Dirichlet Regression
Source: PLoS One. 2015 Jun 26;10(6):e0129341. doi: 10.1371/journal.pone.0129341 (PMC4482627; doi:10.1371/journal.pone.0129341)
Supplement: S2 Text — (DOC) [file pone.0129341.s005.doc]

**S3 Text: Study organisms and experimental details.**

All species of terrestrial and aquatic insects used in the experiments are common and widespread in Europe or the Palaearctic. *Amara communis* (adult body length 6–8 mm) inhabits the turf layer in open grassy habitats. Its larvae and adults are omnivores that feed mainly on seeds but also may consume small invertebrates; pupation occurs in the soil. Both leaf beetle species used in this study are open feeders at the larval and adult stages and heavily damage leaves of their host plants: *Gastrophysa* *viridula* (body length 4–6 mm) feeds on *Rumex* spp. and *Leptinotarsa decemlineata* (body length 9–12 mm) is a notorious potato pest; both species pupate in the soil. *Loxostege sticticalis* (wing span 20–26 mm) is an outbreaking migratory species of moth; larvae feed on a wide range of plants and pupate in web cocoons. *Microvelia* *reticulata* (body length 1.4–1.6 mm) colonizes still water, while *Velia caprai* (body length 7–8 mm) occurs along forest streams [1,2]. *Notonecta glauca* (body length 14–16 mm) is found in a wide variety of standing water habitats [3]. All three species develop through five larval instars (L1–L5) before moulting into an adult. *Acilius canaliculatus* (body length 14–16 mm) lives in standing waters and the adult is preceded by three larval instars (L1–L3) and a pupa. Finally, *Cloeon dipterum* (body length 6–10 mm) is polyvoltine and has, as most mayflies, highly variable number of instars and one final preimaginal stage, the subimago [4].

*Experimental design*

Data for all species were obtained in rearing experiments carried out at the Institute of Entomology of the Biology Centre CAS, vvi, České Budějovice, Czech Republic (*Acilius*, *Cloeon*), at the Faculty of Education of the University of South Bohemia in České Budějovice (*Microvelia*, *Velia*, *Notonecta*), and at St. Petersburg State University, Laboratory for Evolutionary and Physiological Ecology of Insects, Stary Peterhof, Russia (*Amara*, *Gastrophysa*, *Leptinotarsa*, *Loxostege*).

Adults of all terrestrial species were collected in the field in spring and kept in pairs or groups in glass or plastic vials. Eggs were collected once a day (*Amara*, *Loxostege*) or twice a day (*Gastrophysa*, *Leptinotarsa*) and reared individually (*Amara*) or in clutches (*Gastrophysa*, *Leptinotarsa*, *Loxostege*). Immediately after collection, Petri dishes with eggs were allocated among several environmental chambers with constant temperatures and photoperiods (S3 Table). Larvae were reared in Petri dishes (40–100 mm in diameter) and then in larger plastic containers (250 ml volume, approx. 11x8x5 cm in size, *Leptinotarsa*) or glass jars (0.5-1 l volume, *Loxostege*). *Leptinotarsa,* *Gastrophysa* and *Loxostege* were reared in groups that were split in order to maintain optimal density as the larvae grew. *Amara* pupated in moist sand that was kept continuously in the Petri dishes. *Gastrophysa*, *Leptinotarsa* and *Loxostege* were transferred for pupation to containers and jars with sawdust after the cessation of feeding; the sawdust for leaf beetles was wet and that for *Loxostege* was dry. Further details of the rearing methods for *Amara*, *Gastrophysa* and *Loxostege* can be found in earlier papers (*Amara*: [5]; *Gastrophysa*: [6]; *Loxostege*: [7], wherefrom the data for the present study are taken). Rearing of *Leptinotarsa* was similar in all key aspects to that of *Gastrophysa*.

Overwintered females of *Microvelia* and *Notonecta* laid eggs in the laboratory aquaria, which were randomly placed into one of the three temperatures at constant 16L:8D photoperiod. Each nymph was isolated upon hatching and reared in a small plastic cup (~0.1 l volume) with a 10 cm^2^ surface filled with 15–20 ml of aged tap water (*Microvelia*) or in a lager 0.2 l plastic cup filled with ~170 ml of water (*Notonecta*). Water was exchanged once a week or earlier when a bacterial biofilm begun to develop on the water surface.

Repeated attempts to routinely obtain sufficiently large numbers of eggs in the laboratory failed for *Velia*, *Acilius* and *Cloeon*. Thus, early instar larvae were collected in the field and subsequently used in the experiment. First-instar *Velia* nymphs were collected in spring and immediately placed randomly into one of the three rearing temperatures with constant 16L:8D photoperiod (S3 Table). They were reared in plastic cups with about 20 cm^2^ surface area and filled with a small amount of aged tap water that was exchanged once a week or earlier when a bacterial biofilm begun to develop on the water surface. Because the method used for rearing *Microvelia* and *Velia* was nearly identical with a previous experiment [8] that focused on a different question at one particular temperature, we added the data from that paper to our dataset*.*

First-instar larvae of *Acilius* were collected in May 2013 and first kept at a 17.5°C, which was close to the water temperature at the locality around the date of capture (15–18°C) as well as to the midpoint of the temperature range used in the experiments (14–20°C). Upon moulting into L2, they were immediately placed randomly into one of the three rearing temperatures and constant 16L:8D photoperiod (S3 Table). L1 and L2 larvae were first kept in 0.2 l plastic cups with ~170 ml of aged tap water, which was exchanged once every 4 days. Because diving beetles pupate on land and require plant debris to construct a protective pupal chamber, larvae were individually transferred to larger rectangular rearing chambers several days after their moulting into L3; the transfer was timed at approximately the same relative duration of L3 instar in each temperature and hence differed between treatments (after 5 days in 20 °C, 8 days in 17°C and 10 days in 14°C, based on previous pilot experiments). The rearing chamber contained a similar volume of water that was also exchanged daily. The chamber had a gently sloping bottom covering ~80% of the entire length of the 6x20 cm bottom area. The bottom was covered with coarse sand embedded into a synthetic rubber matrix, so that the larva could freely climb out of the water into an attached pupal chamber (6x6x9 cm). The pupal chamber was filled with a 5 cm layer of moist coarse sand and covered with a layer of wet plant debris collected at the same site as the larvae and sterilized by short immersion in boiling water*.* Pupal chambers containing pupae were detached from the rearing chamber and covered by Plexiglas plates to prevent evaporation and escape of the eclosed adults. We maintained ~100% humidity in pupal chambers using a water sprayer.

It was impossible to find first instar nymphs of *Cloeon* in the field or ascertain their instar after they were brought in the laboratory. Thus, similarly sized early- to medium-instar larvae (body size approximately 3 mm) were collected in the field in early August 2012, placed individually in 0.2 l plastic cups with ~140 ml of aged tap water and randomly assigned to one of the two temperature treatments with constant photoperiod 15L:9D (S3 Table). The cups were kept in common aquaria with continuously aerated, aged tap water. Each cup was equipped with two 9-cm^2^ holes cut under the water level and covered with 0.17 mm mesh to allow water circulation; water was changed once a week. A small piece of white Styrofoam (approx 0.5-1 cm^3^) was added to each cup at the end of larval development to provide the adult with a resting place.

Larvae of all species were fed daily ad libitum. *Amara* larvae were given grass seed mixture. *Gastrophysa* was fed with freshly collected *Rumex* leaves which were renewed twice a day for late instars at higher temperatures and once a day in other cases. Similarly, *Leptinotarsa* was supplied with fresh potato twigs and leaves (varieties Nevsky and Charodey) and *Loxostege* was fed with *Chenopodium album* twigs once or twice a day as required. All nymphs of *Microvelia* and L1 and L2 nymphs of *Velia* were fed with one frozen fruit fly (*Drosophila melanogaster*) and later instars of *Velia* with two fruit flies. Zooplankton diet composed mainly of cladocerans (mainly *Daphnia magna*, approx. 300 individuals per day) and copepods was given to *Notonecta* and *Acilius* larvae; L3 *Acilius* larvae were further supplied with two chironomid larvae at 14°C and three chironomid larvae at 17 and 20°C because pilot experiments showed that pure zooplankton diet leads to elevated mortality of L3 larvae (P. Dudová and D.S. Boukal, unpublished data). *Cloeon* nymphs were fed with an excess of *Stigeoclonium* sp. culture and a ~2–3 cm long cut of a dead *Typha* sp. leaf.

*Data collection*

Individuals of terrestrial insects were checked twice a day (at 12-hour intervals); only *Loxostege* oviposition and pupation of all species at the lowest temperatures (18 or 16 °C) were monitored every 24 hours. Moults between subsequent larval instars were not recorded. In all terrestrial beetle species (*Amara*, *Gastrophysa*, and *Leptinotarsa*), durations of the following developmental stages were obtained: egg (from oviposition to hatching), larva (from hatching to pupation, i.e., including the non-feeding prepupal stage), and pupa (from pupation to adult eclosion). *Loxostege* caterpillars spin cocoons before pupation. Exact time of pupation was therefore not determined to avoid damage to the larva inside the cocoon and the immature development of *Loxostege* was divided into the following stages: egg (from oviposition to hatching), larval feeding period (from hatching to cocoon spinning), and prepupa+pupa (period spent inside the cocoon).

In experiments on all aquatic and semiaquatic species, individuals were checked daily at approximately 24 hour intervals (maximum difference of about 1 hour between days) for moulting, indicated by the presence of the exuvium of the preceding instar. This provided detailed data on developmental time (in days) of all larval instars (L1–L5) for *Microvelia*, L2–L5 instars for *Velia* and *Notonecta*, and data on L2, L3 and pupa for *Acilius.* Similar to *Loxostege*, we used the day at which the larva left water as a reliable proxy for the pupation date of *Acilius* in order not to disrupt the larva during pupation; pilot observations suggested that the larvae pupate rapidly after leaving water, usually within a day (P. Dudová and D.S. Boukal, unpublished data). Emergence of adult *Acilius* was observed daily by a careful direct inspection of the pupal chamber; this was carried out daily after a delay of several days during which the adults never hatched (P. Dudová and D.S. Boukal, unpublished data). The newly observed adults were clearly immature as they lacked pigmentation or had very soft cuticle; they were left in the pupal chamber until the next day before they partially hardened and obtained typical adult coloration.

Because mayflies do not have a fixed number of preimaginal developmental stages, matching instars do not have to share the same level of development, especially during early and intermediate ontogeny. However, during the last two larval instars preceding the subimago, the individuals are likely to have developed to similar levels. Development of *Cloeon* was thus divided into a stage containing 1–5 instars from the start of the experiment to the ecdysis into the pre-final instar (hereafter called “early stage”), the pre-final instar, and the final instar before the subimago emerged. We assigned individuals to both temperatures randomly with respect to size and therefore most likely also with respect to developmental stage. Pooling a varying number of instars into the early stage therefore should only introduce additional noise into the data but otherwise do not affect the analyses. Duration of *Cloeon* subimago was not included in the data because the stage lasted less than one day in all treatments and its duration could not be reliably determined.

**References for Appendix S3**

1. Andersen NM. The semiaquatic bugs (Hemiptera, Gerromorpha). Phylogeny, adaptations, biogeography, and classification. Entomonograph. 1982;3: 1–455.

2. Ditrich T, Papáček M, Heino M. The latitudinal uniformity of the unique life history of Velia caprai (Heteroptera: Veliidae) and notes to the pre-overwintering period of selected water striders (Heteroptera: Gerridae). Entomol Fenn. 2011;22: 106–112.

3. Savage AA. Adults of the British aquatic Hemiptera Heteroptera: a key with ecological notes. Ambleside, Cumbria, UK: Freshwater Biological Association; 1989.

4. Brittain JE. Biology of mayflies. Annu Rev Entomol. 1982;27: 119–147.

5. Lopatina EB, Kipyatkov VE, Balashov SV, Kutcherov DA. Photoperiod–temperature interaction – a new form of seasonal control of growth and development in insects and in particular a carabid beetle, Amara communis (Coleoptera: Carabidae). J Evol Biochem Physiol. 2011;47: 578–592.

6. Kutcherov DA, Kipyatkov VE. Control of preimaginal development by photoperiod and temperature in the dock leaf beetle Gastrophysa viridula (De Geer) (Coleoptera, Chrysomelidae). Entomol Rev. 2011;91: 692–708.

7. Kutcherov D, Saulich A, Lopatina E, Ryzhkova M. Stable and variable life-history responses to temperature and photoperiod in the beet webworm Loxostege sticticalis (Lepidoptera: Crambidae). Entomol Exp Appl. 2015;154: 228–241.

8. Ditrich T, Papáček M. Effect of population density on the development of Mesovelia furcata (Mesoveliidae), Microvelia reticulata and Velia caprai (Veliidae) (Heteroptera: Gerromorpha). Eur J Entomol. 2010;107: 579–587.
