# Supplementary material for: Analyses of Developmental Rate Isomorphy in Ectotherms: Introducing the Dirichlet Regression
Source: PLoS One. 2015 Jun 26;10(6):e0129341. doi: 10.1371/journal.pone.0129341 (PMC4482627; doi:10.1371/journal.pone.0129341)
Supplement: S3 Text — (DOC) [file pone.0129341.s006.doc]

**S5 Text: Example of DRI analysis using Dirichlet regression.**

This R script outlines the analysis carried out in the paper, using our data on *Acilius* as an example; see Table S5 for all data.

## example of DRI analysis using Dirichlet regression

library(ggplot2)

library(DirichletReg)

library(plyr)

## set working directory, e.g.:

setwd('C://data/DRI/')

## function to build table with model names, function, AIC and AICc #####

res <- function (models) {

data.frame(cbind(ldply(models, function(x) cbind(AIC = AIC(x), BIC = BIC(x),

AICc = AIC(x) + 2 * x$npar * (x$npar+1) / (nobs(x)-x$npar-1))),

model = sapply(1:length(models), function(x) deparse(formula(models[[x]])))

) ) }

# function for data shifting

negshift <- function(x){c(x[2:(length(x))], NA)}

## data input #########

## duration of each instar followed in the experiment (days)

## tot.dev gives duration of entire development (i.e. sum of durations of all instars)

acil <- read.table("DRI_Acilius.csv", header=TRUE, sep=",")

acil$temp <- as.numeric(acil$temp)

acil$tempF <- as.factor(acil$temp)

acil$drate <- 1/acil$tot.dev

acil$props <- DR_data(acil[, 3:5]) ## calculate proportional duration of L2, L3 and pupa in each individual

## test of linear dependence of development rate on temperature ####

lm.dev <- lm(drate ~ fac*(temp + I(temp^2)), data = acil)

lm.dev <- step(lm.dev)

summary(lm.dev)

## visualize the dependence of development rate on temperature

p <- ggplot(acil, aes(x=temp, y=drate, group = temp))

p + geom_jitter(aes(x=temp, y=drate), alpha=0.4, position=position_jitter(width=0.1)) +

stat_smooth(method=lm, aes(group=1)) + facet_grid(. ~ fac) + theme_bw()

## construct candidate models ####

dr.DRI <- DirichReg(props ~ 1, acil)

dr.fDRI <- DirichReg(props ~ fac, acil)

dr.t <- DirichReg(props ~ temp, acil)

#dr.t2 <- DirichReg(props ~ temp + I(temp^2), acil) ## for three temperatures identical with dr.tF

dr.ft <- DirichReg(props ~ fac + temp, acil)

dr.fti <- DirichReg(props ~ fac * temp, acil)

dr.ftFi <- DirichReg(props ~ fac * tempF, acil)

#dr.ft2 <- DirichReg(props ~ fac + temp + I(temp^2), acil) ## for three temperatures identical with dr.ftF

dr.tF <- DirichReg(props ~ tempF, acil)

dr.ftF <- DirichReg(props ~ fac + tempF, acil)

#### compare models

models <- list(dr.DRI, dr.fDRI, dr.t, #dr.t2, dr.ft, dr.fti, dr.ftFi, # dr.ft2, dr.tF, dr.ftF)

## rank models by increasing AICc values

comp.Dir <- res(models)

comp.Dir[order(comp.Dir$AICc), ]

## predict values for selected (best) models ####

## values of explanatory variables

pred.grid <- expand.grid(fac= sort(unique(acil$fac)), temp =sort(unique(acil$temp)))

pred.gridF <- expand.grid(fac= sort(unique(acil$fac)), tempF =sort(unique(acil$tempF)))

## best model for Acilius

pred.ftF <- cbind(pred.gridF, predict(dr.ftF, pred.gridF))

colnames(pred.ftF)[3:5] <- c("L2", "L3", "pupa")

print(pred.ftF, digits = max(3, getOption("digits") - 3))

## second best model for Acilius

pred.ft <- cbind(pred.grid, predict(dr.ft, pred.grid))

colnames(pred.ft)[3:5] <- c("L2", "L3", "pupa")

print(pred.ft, digits = max(3, getOption("digits") - 3))

## third best model for Acilius

pred.tF <- cbind(pred.gridF, predict(dr.tF, pred.gridF))

colnames(pred.tF)[3:5] <- c("L2", "L3", "pupa")

print(pred.tF, digits = max(3, getOption("digits") - 3))

## fourth best model for Acilius

pred.t <- cbind(pred.grid, predict(dr.t, pred.grid))

colnames(pred.t)[3:5] <- c("L2", "L3", "pupa")

print(pred.t, digits = max(3, getOption("digits") - 3))

## slopes in the Dirichlet model with temperature+sex dependence

## (the same procedure applies to prediction of the model with temperature dependence, pred.t)

## calculate slopes between two successive temperatures for each stage and factor level

pred <- ddply(pred.ft, .(fac), transform,

nx.t = negshift(temp),

nx.L2 = negshift(L2),

nx.L3 = negshift(L3),

nx.pa = negshift(pupa) )

pred <- ddply(pred, .(temp, fac), summarize,

sl.L2 = (nx.L2 - L2)/(nx.t - temp),

sl.L3 = (nx.L3 - L3)/(nx.t - temp),

sl.pa = (nx.pa - pupa)/(nx.t - temp) )

## DRI violation slope in %.(degC)^-1: slopes averaged over all temperatures and factor levels

(tslope <- ddply(pred, .(), summarize,

sl.L2 = 100 * mean(sl.L2, na.rm=TRUE),

sl.L3 = 100 * mean(sl.L3, na.rm=TRUE),

sl.pa = 100 * mean(sl.pa, na.rm=TRUE)) )

## DRI violation slope in %.(degC)^-1: factor-specific slopes, averaged over all temperatures

(tslope <- ddply(pred, .(fac), summarize,

sl.L2 = 100 * mean(sl.L2, na.rm=TRUE),

sl.L3 = 100 * mean(sl.L3, na.rm=TRUE),

sl.pa = 100 * mean(sl.pa, na.rm=TRUE)) )

### end of example
